# Intratumoral Injection of Engineered *Mycobacterium smegmatis* Induces Antitumor Immunity and Inhibits Tumor Growth

**DOI:** 10.34133/bmr.0130

**Published:** 2024-01-07

**Authors:** Hang Zhou, Junmeng Zhu, Yi Mei, Aoxing Chen, Rui Liu, Xiaonan Wang, Xiangyu Wu, Xiaotong Chen, Baorui Liu

**Affiliations:** ^1^ The Comprehensive Cancer Centre, Nanjing Drum Tower Hospital, Affiliated Hospital of Medical School, Nanjing University, Nanjing 210008, China.; ^2^ The Comprehensive Cancer Centre of Nanjing Drum Tower Hospital, Clinical College of Traditional Chinese and Western Medicine, Nanjing University of Chinese Medicine, Nanjing 210008, China.; ^3^ The Comprehensive Cancer Centre, China Pharmaceutical University Nanjing Drum Tower Hospital, Nanjing 210008, China.

## Abstract

Conventional type 1 dendritic cells are essential for antigen presentation and successful initiation of antitumor CD8^+^ T cells. However, their abundance and function within tumors tend to be limited. *Mycobacterium smegmatis*, a fast-growing, nonpathogenic mycobacterium, proves to be easily modified with synthetic biology. Herein, we construct an engineered *M. smegmatis* expressing a fusion protein of Fms-like tyrosine kinase 3 ligand and costimulator CD40darpin (rM-FC) since the 2 drugs are reported to have a good synergistic effect. Intratumoral delivery of rM-FC effectively recruits and activates dendritic cells (DCs), especially CD103^+^ DCs and CD80^+^CD86^+^ DCs, further inducing sufficient migration of effector memory T cells into the tumor microenvironment. This successfully converts the so-called immune-desert tumors to the “hot” phenotype. In B16F10 mouse melanoma tumor models, local injection of rM-FC into the primary tumor triggers a robust T cell immune response to restrain the growth of both the treated tumors and the distant untreated ones. The population of PDL1^+^ tumor cells increased after the in situ vaccination, and murine tumors became more responsive to programmed death ligand 1 (PDL1) blockade, prompting the combination therapy. Overall, our findings demonstrate that rM-FC acts as a strong DC agonist and remarkably enhances antitumor immunity.

## Introduction

Traditional treatments, including surgery, chemotherapy, and radiotherapy, often struggle to achieve complete eradication of cancer [1,2]. The ongoing development of immunotherapy has revolutionized the cancer treatment paradigm [3–6]. However, several challenges persist, including high toxicity to normal cells, inability to infiltrate deep tumor tissues, and the recurrence of tumors, necessitating the exploration of alternative strategies. With the wide application of synthetic biology, bacteria-based therapies are considered a promising strategy [7–11]. As early as the 1890s, William Coley used a heat-inactivated mixture of bacteria (Coley’s toxin) to treat tumors and observed tumor ablation. Afterward, Bacille Calmette–Guérin (BCG), a live attenuated strain of *Mycobacterium bovis*, has been used as an effective immunotherapy for the treatment of superficial bladder cancer due to its inherent immunostimulatory efficacy [12]. Considering the biological particularity of bacteria and the fast development of synthetic biology, it has become a unique research field to design immunotherapies using bacteria as carriers [13–17].

Previous studies have shown that intratumoral injection of Fms-like tyrosine kinase 3 ligand (Flt3L) has a good synergistic effect with CD40 agonists [18,19]. CD40darpin, constructed by our laboratory, is a multivalent tandem CD40-targeting protein acting as a CD40 agonist. Although intratumoral administration can focus the activity of immunostimulatory agents in tumors while lowering systemic toxicity, providing a potential strategy to induce T cell inflammation and converting the “cold” immune inert tumor microenvironment (TME) into a “hot” immune inflamed TME [20–22], it is difficult to ensure the persistence of small-molecule drugs in the TME since free drugs are easily cleared via lymphatics and/or vasculature. Bacteria offer the benefits of serving as natural vectors for the sustained release of therapeutic agents while simultaneously functioning as immune-activating agents. *Mycobacterium smegmatis*, a nonpathogenic member of the *Mycobacterium* family, outpaces BCG in growth and is a saprophytic environmental mycobacterium derived from soil. Its ease of genetic modification through synthetic biology techniques renders it an ideal vaccine carrier [23–27]. Unlike BCG, *M. smegmatis* acts as an immune adjuvant to promote cellular immune responses [28,29]. It can induce macrophages to release cytokines [30] and better induce the maturation of dendritic cells (DCs) [31].

**Fig. 1.**
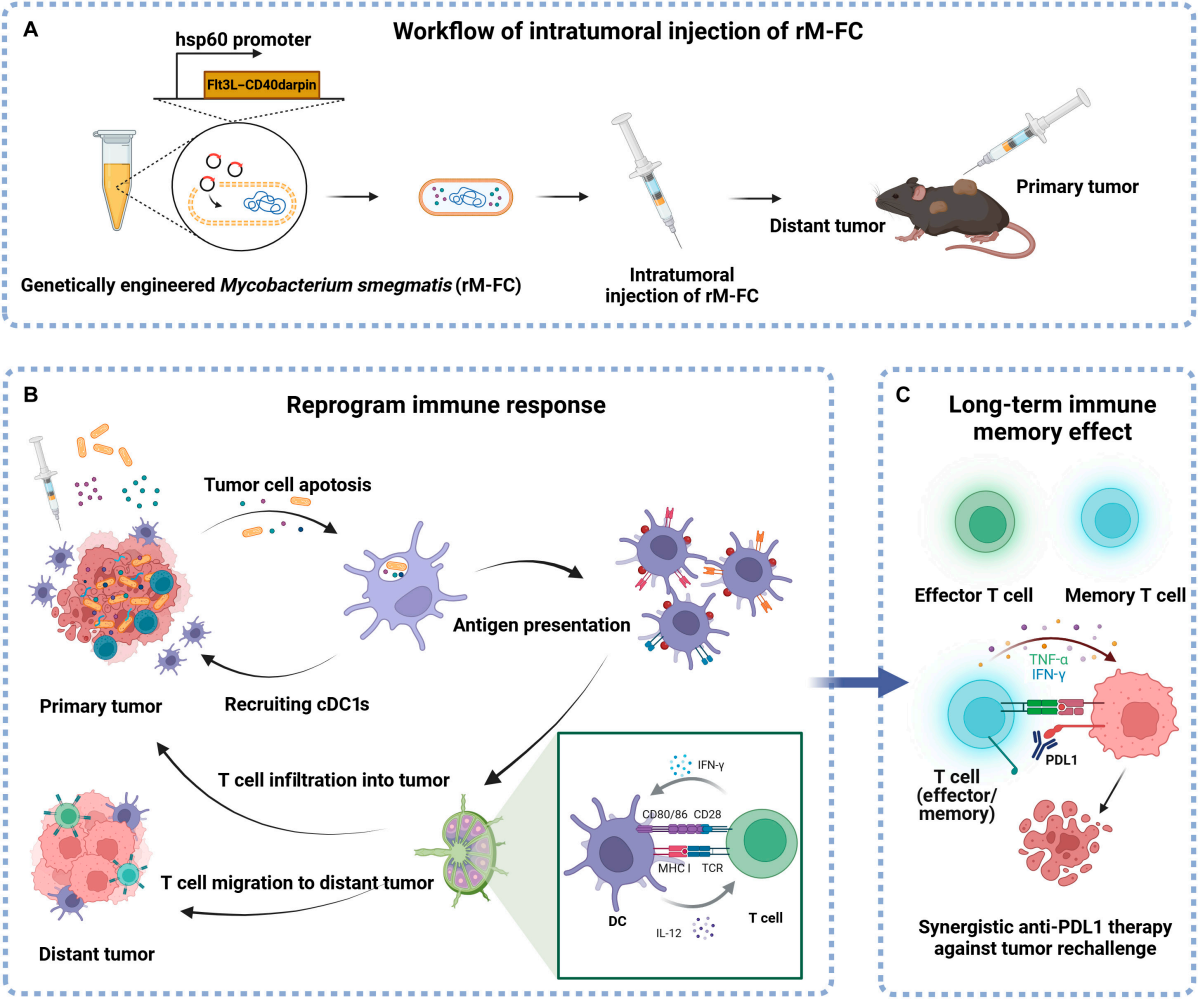
Antitumor immune activation and regulatory mechanisms of recombinant *Mycobacterium smegmatis* expressing a fusion protein of human Fms-related tyrosine kinase 3 ligand (hFlt3L)–human CD40darpin (hCD40darpin) (rM-FC). (A) Schematic diagram of the engineered recombinant *M. smegmatis*. Briefly, the Fms-like tyrosine kinase 3 ligand (Flt3L)–CD40darpin gene fragment was cloned into a bacterial expression vector, and after induced protein expression, mice were treated by intratumoral injection. (B) Engineered *M. smegmatis* (rM-FC) induces a strong antitumor immune response and remodels the tumor microenvironment (TME), which combats the growth of distant tumors. Briefly, Flt3L could recruit conventional type 1 dendritic cells (cDC1s) into the TME and CD40darpin could further activate dendritic cells (DCs). *M. smegmatis* itself could lead to tumor cell lysis and upgrade the expression of CD40 on DCs in the TME after intratumoral injection. Flt3L, CD40darpin, and *M. smegmatis* have strong synergistic effects. (C) Engineered *M. smegmatis* synergistic anti-programmed death ligand 1 (anti-PDL1) therapy and generates long-term immune memory, which is against tumor rechallenge. IFN-γ, interferon-γ; MHC I, major histocompatibility complex class I; IL-12, interleukin 12; TNF-α, tumor necrosis factor-α.

Based on the above advantages of *M. smegmatis*, we designed an engineered *M. smegmatis* with the plasmid encoding the fusion protein of Flt3L and costimulator CD40darpin (rM-FC). The 3 main elements, namely, *M. smegmatis*, Flt3L, and CD40darpin, can induce strong stimulation on DCs. Intratumoral administration of rM-FC could effectively suppress the growth of CT26 tumors and B16F10 tumors, prolonging the survival period. Notably, we revealed that rM-FC could upregulate the number of mature dendritic cells in tumor-draining lymph nodes (TDLNs), activate intratumoral effector T cells, increase the presence of CD103^+^ DCs within the treated tumor, facilitate the transition of anti-inflammatory macrophages (M2) to pro-inflammatory macrophages (M1), and upgrade the expression of various effector cytokines (Fig. 1). On top of it, rM-FC could exert a distant effect and synergize with anti-programmed death ligand 1 (anti-PDL1) antibodies, stimulating strong immune memory to reject tumor rechallenge. Overall, our findings illustrated the promise of utilizing rM-FC for in situ vaccination to remodel the TME, suppress tumor growth, and deter tumor recurrence.

## Materials and Methods

### Bacterial culture, cell lines, and mice

*M. smegmatis* mc^2^155 was obtained from the American Type Culture Collection (USA), and pMV261 vector was obtained from Miaolingbio (Wuhan, China). *Escherichia coli* BL21 (DE3) was purchased from Shanghai Weidi Biotechnology Co, Ltd. For bacterial culture, *E. coli* were incubated in Luria–Bertani medium at 37 °C with shaking at 220 rpm. *M. smegmatis* mc^2^155 were grown in Middlebrook 7H9 broth (Solarbio) supplemented with 10% ADC (5% bovine serum albumin, 2% dextrose, and 0.003% catalase) in a shaking incubator (37 °C, 200 rpm). CT26 colon cancer cells and B16F10 melanoma cells were obtained from the Cell Bank of Shanghai Institute of Biochemistry and Cell Biology. CT26 cells, B16F10-OVA, and B16F10 melanoma cells were cultured in RPMI 1640 medium (Gibco) containing 10% fetal bovine serum (FBS), 100 U ml^−1^ penicillin, and 100 μg ml^−1^ streptomycin at 37 °C under an atmosphere of 5% CO_2_. BALB/c and C57BL/6 mice aged 5 to 6 weeks were purchased from Shanghai Sippr-BK Laboratory Animal Co. Ltd. (Shanghai, China). All mice were kept in the specific pathogen-free Laboratory Animal Center of Affiliated Nanjing Drum Tower Hospital of Nanjing University Medical School (Nanjing, China). All animal experimental protocols were approved by the Laboratory Animal Care and Use Committee of the Affiliated Nanjing Drum Tower Hospital of Nanjing University Medical School.

### Preparation and characterization of CD40darpin and rM-FC

Flt3L–CD40darpin was designed in the following orientation: extracellular domain of Flt3L–GGGGSGGGGSGGGGSGGGGS–CD40darpin. The DNA fragments were synthesized, cloned into a bacterial expression vector pET28a or pMV261 by ICarTab Biomedical Co., Ltd. (Suzhou, China), and confirmed by enzyme digestion and DNA sequencing.

CD40darpin protein was expressed in *E. coli* BL21 (DE3) after induction by isopropyl β-d-1-thiogalactopyranoside. Bacterial cultures were harvested by centrifugation, resuspended, and disrupted by sonication. The products were subjected to 12% sodium dodecyl sulfate–polyacrylamide gel electrophoresis (SDS-PAGE) analysis for confirmation. The CD40darpin protein was purified by an ÄKTA system HisTrap HP column (GE HealthCare, CT, USA) according to the manufacturer’s instructions. Protein concentrations were determined using a bicinchoninic acid assay kit (Thermo Fisher Scientific, Massachusetts, USA).

For rM-FC production, *M. smegmatis* mc^2^155 was incubated at 37 °C with shaking at 200 rpm for about 2 d and then transferred to a fresh medium at a ratio of 1:100. When the optical density (OD) value reached 0.6 to 0.8, we placed it on ice for 2.5 h. The above pre-cooled bacteria were subsequently collected by centrifugation (5,000 rpm, 4 °C, and 15 min), and the bacterial pellet was washed 3 times with 10% pre-chilled glycerol and finally resuspended with 10% glycerol to obtain competent bacteria. The pMV261 recombinant plasmid containing the Flt3L–CD40darpin fusion gene was electroporated into competent bacteria at 2.5 kV, 25 μF, and 1,000 Ω (Bio-Rad, USA) at 4 °C. Transformants were selected on solid Middlebrook 7H10 agar plates (Hopebio) containing 50 μg ml^−1^ kanamycin at 37 °C. Transformed bacterium clones from the plates were transferred into Middlebrook 7H9 liquid broth medium with 50 μg ml^−1^ kanamycin at 37 °C with shaking at 200 rpm. Two days later, they were transferred to a fresh liquid medium containing kanamycin at a ratio of 1:100. When the OD value reached 0.6 to 0.8, the expressed protein was induced by heat shock at 45 °C for 2 h. The culture was harvested by centrifugation (5,000*g*, 4 °C, and 15 min). Ultrasonic disruption (Sonics VCX 130, USA; 130 W and 20 kHz) of the obtained bacterial pellet lasted for 6 min (40% amplitude, 5 s, 5 s), and bacterial lysates were collected by centrifugation (12,000*g*, 4 °C, and 15 min). The supernatant of the lysates was mixed with 5× loading buffer in a 4:1 ratio and subjected to Western blot (WB) using an anti-Flt3L antibody (Abcam ab52648). rM-F and rM-C were constructed similarly to rM-FC.

### In vitro bone-marrow-derived cells’ uptake of rM-FC

Bone-marrow-derived cells (BMDCs) derived from C57BL/6 mice were cultured with RPMI 1640 medium containing 10% FBS and 1% penicillin–streptomycin as reported in the previous literature [32]. We added 20 ng ml^−1^ recombinant murine granulocyte–macrophage colony-stimulating factor (Xiamen Amoytop Biotech Co., Ltd., China) and 10 ng ml^−1^ recombinant murine interleukin 4 (PeproTech, USA) into the medium to induce the cells to differentiate into DCs. During cell culture, we replaced the medium every 3 d and collected all cells for use on day 8. DiO (Bridgen, Beijing, China)-stained rM-FC were coincubated with DiI (Bridgen, Beijing, China)-stained DCs for 2 h at 37 °C in the dark. Then, we assessed them by flow cytometry (Beckman Coulter, Germany) or imaged them under a confocal laser scanning microscope (Leica, Germany) to analyze the process of internalization into DCs.

### In vitro BMDC stimulation

BMDCs obtained in vitro were co-cultured with different concentrations of M (*M. smegmatis*) and rM-FC for 20 h. Then, DCs were collected by centrifugation at 300*g* for 5 min and incubated with fluorescein isothiocyanate (FITC)–CD11c, APC–CD80, and phycoerythrin (PE)–CD86 for 30 min before evaluation.

### Near-infrared imaging of BALB/c mice

rM-FC (1 × 10^8^ colony-forming units [CFU]) and DiR dye (Bridgen, Beijing, China) were incubated at 37 °C and 5% CO_2_ for 30 min for staining. After centrifugation at 10,000*g* for 10 min, we removed the supernatant and resuspended the pellet in phosphate-buffered saline. This step was repeated 3 times. DiR-labeled rM-FC were injected intratumorally at CT26 tumor-bearing mice. Mice were anesthetized with isoflurane and imaged by an optical and x-ray small-animal imaging system (IVIS Lumina, PerkinElmer, Germany) at 24, 48, 72, 168, and 360 h, respectively. Then, mice were sacrificed and the heart, liver, spleen, lung, kidney, and TDLNs as well as tumors were excised and imaged. For image analysis, the region of interest was used to delineate specific areas to obtain the average fluorescence intensity.

### Bacterial colonization in vivo

After intratumoral injection of rM-FC, the main organs, blood, and tumor tissues of mice were removed at the specified time points. Samples were weighed under sterile conditions and homogenized with phosphate-buffered saline, which were diluted and incubated in solid Middlebrook 7H10 plates at 37 °C for about 4 d. We then counted the bacteria and plotted the curve with CFU per gram as the standard.

### Cytotoxicity assay of mouse splenocytes

The splenocytes of mice in both the normal saline (NS) group and rM-FC (1 × 10^8^ CFU) groups were incubated with B16F10-luc cells in vitro at effector-to-target ratios (E:T) of 5:1, 10:1, and 20:1 at 37 °C and 5% CO_2_. Luminescence was detected by the addition of d-luciferin to B16F10-luc after incubation with splenocytes for 24 h. Cytotoxicity = (positive − experiment)/positive.

### Animal experiments

CT26 tumor cells (1 × 10^6^) were subcutaneously injected in 100 μl of NS on the left lower abdomen of BALB/c mice. When the tumor volume was around 75 mm^3^, CT26 tumor-bearing mice were randomly divided into 4 groups (*n* = 6) and received various treatments once every 2 d, totaling 3 times as follows: group 1, 100 μl NS; group 2, 1 × 10^8^ CFU native *M. smegmatis* mc^2^155 (M); group 3, 30 μg of Flt3L + 50 μg of CD40darpin (F+C); and group 4, 1 × 10^8^ CFU rM-FC (rM-FC). All drugs were dissolved in 100 μl of NS. Tumor volume was monitored every 2 to 3 d and calculated by the following equation: *V* = length × width × width/2. The maximum tumor burden permitted was 1,500 mm^3^. In some cases, this limit was exceeded by the last day of measurement and the mice were immediately euthanized.

For the dose-fumbling experiment of rM-FC, different concentrations of rM-FC were injected intratumorally. Briefly, 1 × 10^5^ B16F10 melanoma cells were subcutaneously injected into the left lower abdomen of C57BL/6 mice. When the tumor size was almost 50 mm^3^, the same treatment condition was applied for B16F10 tumor-bearing mice.

To evaluate the abscopal effect, 2 tumor models were established. The same number of cells (1 × 10^5^ B16F10) was inoculated on the contralateral abdomen of mice 3 d after the primary tumor. Bilateral B16F10 tumor-bearing C57BL/6 mice were randomly divided into 4 groups (*n* = 6) and their primary tumors were treated intratumorally with NS, M, F+C, or rM-FC when the primary tumor size reached about 50 mm^3^.

For anti-PDL1 immunotherapy, B16F10 tumor-bearing mice were randomly divided into 4 groups (*n* = 6) as follows: group 1, NS; group 2, anti-PDL1; group 3, rM-FC; and group 4, rM-FC + anti-PDL1. The anti-PDL1 (200 μg/mouse) was intraperitoneally injected every 3 d after the first injection of rM-FC 3 times.

To evaluate the potency of such a combination effect (rM-FC + anti-PDL1) in blocking tumor recurrence, B16F10 tumor-bearing mice cured by intratumoral injection with rM-FC + anti-PDL1 (*n* = 4) were rechallenged by subcutaneously injecting with 1 × 10^5^ B16F10 cells at 90 d post the first-round treatment. Meanwhile, healthy mice of the same age also received the same dose of tumor inoculation as control.

### Flow cytometry

The following monoclonal antibodies were purchased from BioLegend: CD11c–FITC, CD80–APC, CD86–PE, CD40–PE/Cy5.5, major histocompatibility complex class I (MHC I)–PE/Cy5, MHC II–APC, CD103–PE, CD3–FITC, CD8–APC, CD44–PE, CD62L–PE/Cy7, programmed death 1 (PD1)–APC, PDL1–PE, CD11b–FITC, F4/80–PE/Cy5, CD206–APC, interferon-γ (IFN-γ)–APC, and H-2K^b^ bound to SIINFEKL–PE. Anti-CD8 (mouse) monoclonal antibody–Alexa Fluor 647 (KT15) was obtained from MBL. All antibodies were diluted to 1:100. H-2K^b^ ovalbumin (OVA) tetramer SIINFEKL (TS-5001-1C) was purchased from MBL and used according to the instructions.

On the 10th day after the last treatment, the tumors, spleens, and TDLNs were removed from the mice to collect single-cell suspensions for flow cytometry analysis. The tumor tissues minced into small pieces were digested with collagenase type IV (1 mg ml^−1^, Sigma) for 2 h at 37 °C with gentle agitation, while we obtained spleens and TDLN cell suspensions by mechanical grinding. All samples were then resuspended in NS, stained with specific antibodies for 20 min in 4 °C in the dark, and washed before analysis. LEGENDplex MU Th1/Th2 Panel (8-plex) w/VbP V03 (BioLegend) was used to detect and analyze the level of cytokines in the tumor cell supernatant.

### Histology safety analysis

C57BL/6 mice were sacrificed on the 10th day after the last treatment. Then, the major organs, including hearts, livers, spleens, lungs, and kidneys, were dissected and fixed in 4% formaldehyde. Paraffin-embedded slides were prepared and stained with hematoxylin and eosin for histological analysis under optical microscopy (DM5000, Leica, Germany).

### Statistical analysis

GraphPad Prism 8.0.1 (San Diego, CA, USA) was used for statistical analyses. All data are presented as mean ± standard error of the mean (SEM). Two-way analysis of variance (ANOVA) and Tukey posttest and correction as indicated were used to compare antitumor effects. Two-tailed unpaired Student *t* tests were used to determine the differences in previous data between the 2 groups. For survival studies, log-rank (Mantel–Cox) tests were used. Flow cytometry data were collected with Beckman Coulter (Germany) and cells were analyzed on CytoFLEX (Beckman Coulter) and FlowJo X (FlowJo). *P* < 0.05 was considered having statistical significance (ns means *P* > 0.05, *P < 0.05, **P < 0.01, ***P < 0.001, and ****P < 0.001). Figures were designed in Adobe Illustrator.

## Results

### Construction of the hFlt3L–hCD40darpin fusion protein and the CD40darpin protein

**Fig. 2.**
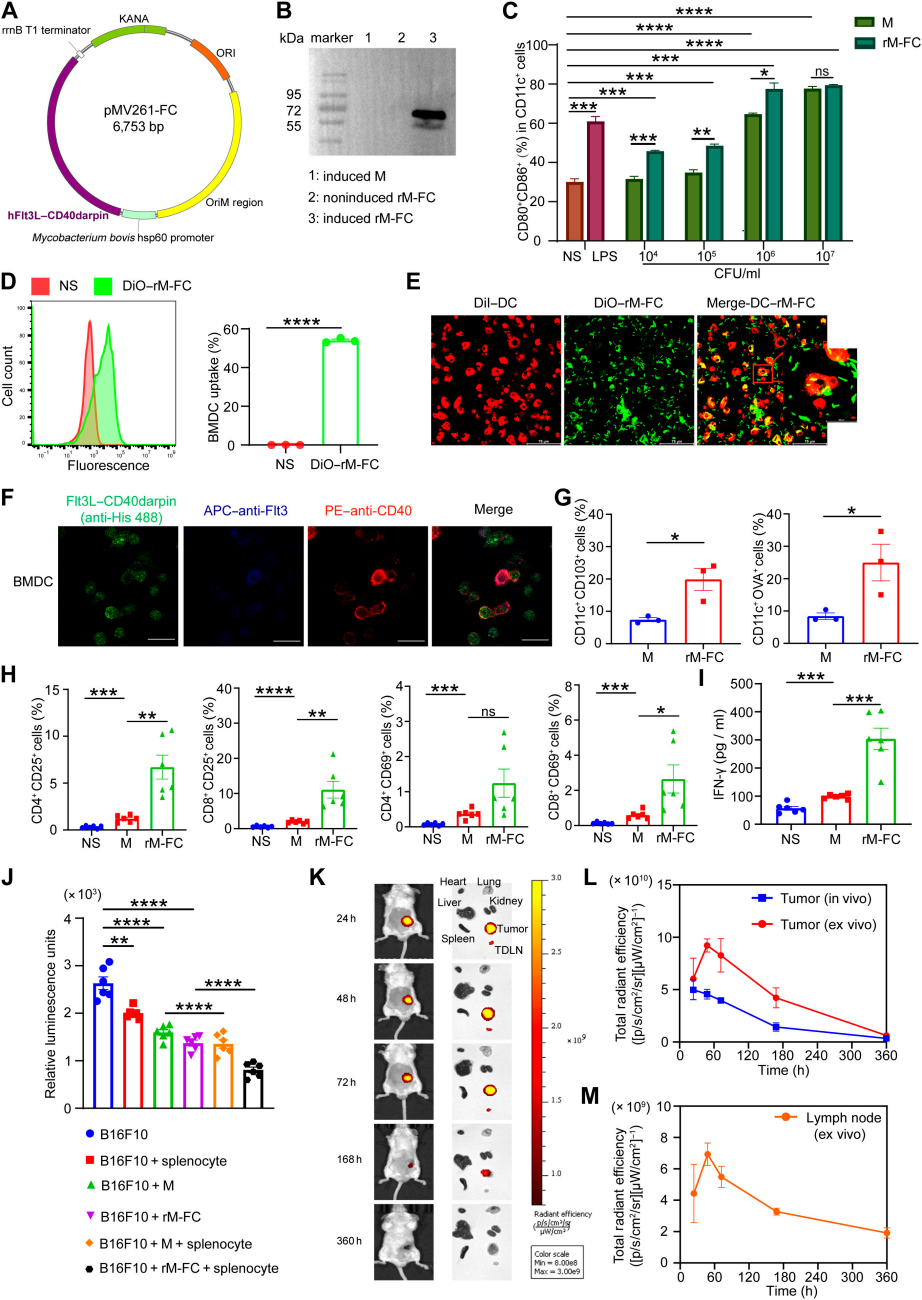
In vitro DC and T cell activation and biodistribution analysis of rM-FC. (A) Plasmid map of a *Mycobacterium*–*Escherichia coli* shuttle vector using pMV261 expressing the hFlt3L–hCD40darpin fusion protein. (B) Western blotting analysis of the induced engineered *M. smegmatis* (rM-FC). Lane marker: molecular mass marker; lane 1: whole bacteriological lysate of wild-type *M. smegmatis* (M) after heat shock; lane 2: rM-FC without heat shock; lane 3: rM-FC induced by heat shock. (C) The percentage of mature dendritic cells (mDCs; CD11c^+^CD80^+^CD86^+^) after coincubation with different concentrations of M or rM-FC in vitro for 20 h (*n* = 3). (D) Cellular uptake of DiO-labeled rM-FC after 2 h of incubation with bone-marrow-derived dendritic cells (BMDCs), as assessed by flow cytometry (*n* = 3). (E) Colocalization analysis of rM-FC (DiO, green) in BMDCs (DiI, red) by confocal microscopy (coincubated for 2 h). (F) The Flt3L–CD40darpin–His fusion protein was extracted from the lysates of rM-FC. Confocal laser scanning microscopy images of mouse BMDCs treated with the Flt3L–CD40darpin fusion protein, allophycocyanin (APC)–antimouse Fms-like tyrosine kinase 3 (Flt3), and phycoerythrin (PE)–antimouse CD40 for 2 h. White scale bars, 40 μm. (G) BMDCs were cultured in RPMI 1640 medium (Gibco) containing 10% fetal bovine serum (FBS), with 20 ng/ml granulocyte–macrophage colony-stimulating factor (GM-CSF) and lysates of 10^7^ colony-forming units (CFU) M/rM-FC added on day 0, day 3, day 5, and day 7. On day 8, 10 nmol ovalbumin (OVA) peptides were added to the medium and coincubated with BMDCs for 24 h. CD11c^+^CD103^+^ DCs and CD11c^+^OVA^+^ DCs were tested on day 9 by flow cytometry (*n* = 3). (H) Splenocytes from C57BL/6 mice were incubated with 10^4^ CFU M or 10^4^ CFU rM-FC for 24 h. The quantification of CD69 and CD25 expression on CD8^+^ and CD4^+^ T cell subsets in the T cells (*n* = 6). (I) Assessment of IFN-γ in co-culture supernatants after splenocytes from C57BL/6 mice stimulated by M or rM-FC for 24 h in vitro (*n* = 6). (J) Luminescence was detected by the addition of d-luciferin to B16F10-Luc cells after incubation with splenocytes, M, and rM-FC for 24 h (*n* = 6). (K) Representative in vivo near-infrared imaging of tumor-bearing mice at 24, 48, 72, 168, and 360 h after intratumoral injection of 10^8^ CFU rM-FC (*n* = 3). (L) Total radiant efficiency of the rM-FC signal in tumors in vivo or ex vivo over time (*n* = 3). (M) Total radiant efficiency of the rM-FC signal in tumor-draining lymph nodes (TDLNs) ex vivo over time (*n* = 3). For the experiments in (C), (D), and (G) to (J), the error bars represent mean ± standard error of the mean (SEM). *P* values were calculated by 2-tailed unpaired Student *t* tests. ns represents *P* > 0.05, * represents *P* < 0.05, ** represents *P* < 0.01, *** represents *P* < 0.001, and **** represents *P* < 0.0001. For the experiments in (L) and (M), the error bars represent mean ± SEM. ORI, origin of replication; KANA, kanamycin; LPS, lipopolysaccharides.

*E. coli*–mycobacterium shuttle vector pMV261 was a plasmid with medium copy numbers, which used the BCG hsp60 promoter for constitutive expression of recombinant proteins. In this study, pMV261 was used as an expression vector for the generation of rM-FC, which could reprogram the TME and enhance the antitumor effect (Fig. 2A). When the OD value of rM-FC was about 0.6 to 0.8, the expression of the human Fms-related tyrosine kinase 3 ligand (hFlt3L)–human CD40darpin (hCD40darpin) fusion protein was induced following heat induction at 45 °C for 2 h. Protein expression was detected by WB assay after lysis of cultured rM-FC. The results revealed that samples from the rM-FC lysate showed specific expression bands with a molecular weight of approximately 72 kDa (lane 3), which was absent in the induced wild *M. smegmatis* (M) (lane 1) and noninduced rM-FC (lane 2) (Fig. 2B). *E. coli* BL21 (DE3) was used to express CD40darpin after induction by isopropyl β-D-1-thiogalactopyranoside. Then, the bacteria were centrifuged and disrupted by sonication. Lysates from cultured bacteria were used for SDS-PAGE to verify protein expression. The results showed that the molecular weight of CD40darpin was about 54 kDa. Metal affinity chromatography was used for the purification of CD40darpin by the ÄKTA fast protein liquid chromatography system. Purified fractions were collected and verified by SDS-PAGE analysis, further confirming the expected molecular size (Fig. S1).

### In vitro DC and T cell maturation triggered by rM-FC

DCs play a vital role in the initiation and regulation of systemic immune responses and are required for T-cell-mediated antitumor immunity. Several studies have shown that *M. smegmatis* plays an important role in DC maturation [31,33]. To evaluate the activation effect of rM-FC on DCs, various concentrations of rM-FC were coincubated with BMDCs in vitro for about 20 h. Increased expression of costimulatory molecules CD80 and CD86 implies that both rM-FC and wild *M. smegmatis* (M) could stimulate DC maturation. We observed that within the concentration range of 10^4^ to 10^6^ CFU ml^−1^, the proportion of mature BMDCs (CD11c^+^CD80^+^CD86^+^) was much higher in the rM-FC group than in the NS group or M group (Fig. 2C). Interestingly, we found that the ability of rM-FC to stimulate DC maturation was markedly enhanced in a dose-dependent manner. When the rM-FC concentration reached 10^6^ CFU ml^−1^, its ability to promote DC maturation was even better than that of lipopolysaccharides. As the concentration increased to 10^7^ CFU ml^−1^, the capacity of rM-FC to induce DC maturation did not differ significantly from that of M. We speculated that DC maturation had reached saturation. We further examined the endocytosis of DCs on rM-FC. rM-FC and DCs were stained with DiO and DiI dyes, respectively, and then incubated at a ratio of 10:1 for 2 h at 37 °C in the dark. Flow cytometry was applied to assess cell-associated fluorescence (Fig. 2D). We observed a marked uptake of rM-FC by BMDCs, which might further induce immune activation. Confocal results also demonstrated the strong capture capacity of DCs to the rM-FC (Fig. 2E). In addition, we extracted the Flt3L–CD40darpin–His fusion protein from lysates of rM-FC and treated BMDCs with anti-His 488, APC–antimouse Fms-like tyrosine kinase 3 (Flt3), and PE-antimouse CD40 for 2 h. It was shown that Flt3L–CD40darpin can bind to Flt3 and CD40 on DCs (Fig. 2F). In order to determine the efficacy of rM-FC for conventional type 1 dendritic cell (cDC1) induction in vitro, we cultured BMDCs in RPMI 1640 medium containing 10% FBS, with 20 ng/ml granulocyte–macrophage colony-stimulating factor and lysates of 10^7^ CFU M/rM-FC added on day 0, day 3, day 5, and day 7. On day 8, 10 nmol of OVA peptides was added to the medium and coincubated with BMDCs for 24 h. We found that the proportions of CD11c^+^CD103^+^ DCs and CD11c^+^OVA^+^ DCs were significantly increased in the rM-FC group than those in the M group (Fig. 2G).

Splenocytes of the C57BL/6 mice were used as a source of DCs and T cells to induce activated T cells. After being treated with live M or rM-FC for 24 h in vitro, both M and rM-FC induced a higher expression of CD69 (early activation marker) than that the NS group. We also observed a higher expression of CD25 (late activation marker) in the rM-FC group than in the M group (Fig. 2H). Consistently, rM-FC promoted the highest level of IFN-γ production (Fig. 2I). To determine whether M and rM-FC could kill cancer cells by direct cell lysis, B16F10-Luc cells were coincubated with M or rM-FC and/or splenocytes in vitro for 24 h. Notably, the luciferase activity of B16F10-luc cells decreased, which demonstrated the lysis of the cancer cells (Fig. 2J).

### In vivo biodistribution of rM-FC

Near-infrared fluorescence imaging was used to observe the distribution of rM-FC in different organs at the indicated time points after intratumoral injection. DiR-labeled rM-FC was injected once to visualize the position of rM-FC. We found that all of the injected rM-FC was mainly trapped in the tumor core (Fig. 2K), and the fluorescence intensity gradually decreased until 360 h later (Fig. 2L). At 24, 48, 72, 168, and 360 h posttreatment, we euthanized the mice and collected their major organs, including hearts, livers, spleens, lungs, kidneys, and TDLNs, as well as the treated tumors for ex vivo. It was found that rM-FC was mainly colonized in the tumors and TDLNs (Fig. 2K), where the fluorescence peaked at 48 h and then gradually weakened until 360 h later (Fig. 2L and M). Overall, the fluorescence intensity of the tumors was stronger than that of the TDLNs. Similarly, we verified the colonization of rM-FC in mice using plate coating. Consistent with the above results, rM-FC was mainly concentrated in the core of the tumors and the TDLNs (Fig. S2). Taken together, these results showed that intratumoral injection of rM-FC could mainly localize inside tumors and TDLNs, indicating that rM-FC had a good biosafety profile.

### Intratumoral injection of rM-FC inhibited tumor growth in the murine CT26 colon tumor model

It was worth noting that we had experimentally proved that Flt3L and CD40darpin had a synergistic antitumor effect, and the combination therapy exhibited a stronger inhibition of tumor growth and a marked extension of animal survival compared to that of any single treatment (Fig. S3A to C). It was found that in situ administration of Flt3L for 3 d increased CD103^+^ DCs in CT26 tumors and TDLNs (Fig. S3D and E), while CD40darpin stimulation increased intratumoral CD86^+^ DCs and CD80^+^CD86^+^ DCs in TDLNs, inducing stronger interleukin 12 (IL-12) and IFN-γ production (Fig. S3F to I).

Based on the result, we constructed rM-FC to evaluate its therapeutic effects. We first conducted dose exploration in male BALB/c mice aged 5 to 6 weeks to determine the optimal dose for intratumoral injection. After inoculating tumors through subcutaneous injection of 1 × 10^6^ CT26 tumor cells into BALB/c mice for 6 d, different concentrations of rM-FC were dissolved in 100 μl of NS. Briefly, we injected 10^6^, 10^7^, 10^8^, and 10^9^ CFU rM-FC intratumorally to explore the antitumor effects. The tumor sizes and body weight of each mouse were monitored after various treatments. We observed that 10^8^ and 10^9^ CFU rM-FC significantly inhibited tumor growth compared to the other 3 groups of NS, 10^6^ CFU, and 10^7^ CFU (Fig. S4). There was no significant weight loss of mice or abnormal death during the treatment within such a wide range of doses of rM-FC. Considering the cost of bacterial preparation and experimentation, we finally adopted 10^8^ CFU/mouse as the therapeutic dose.

**Fig. 3.**
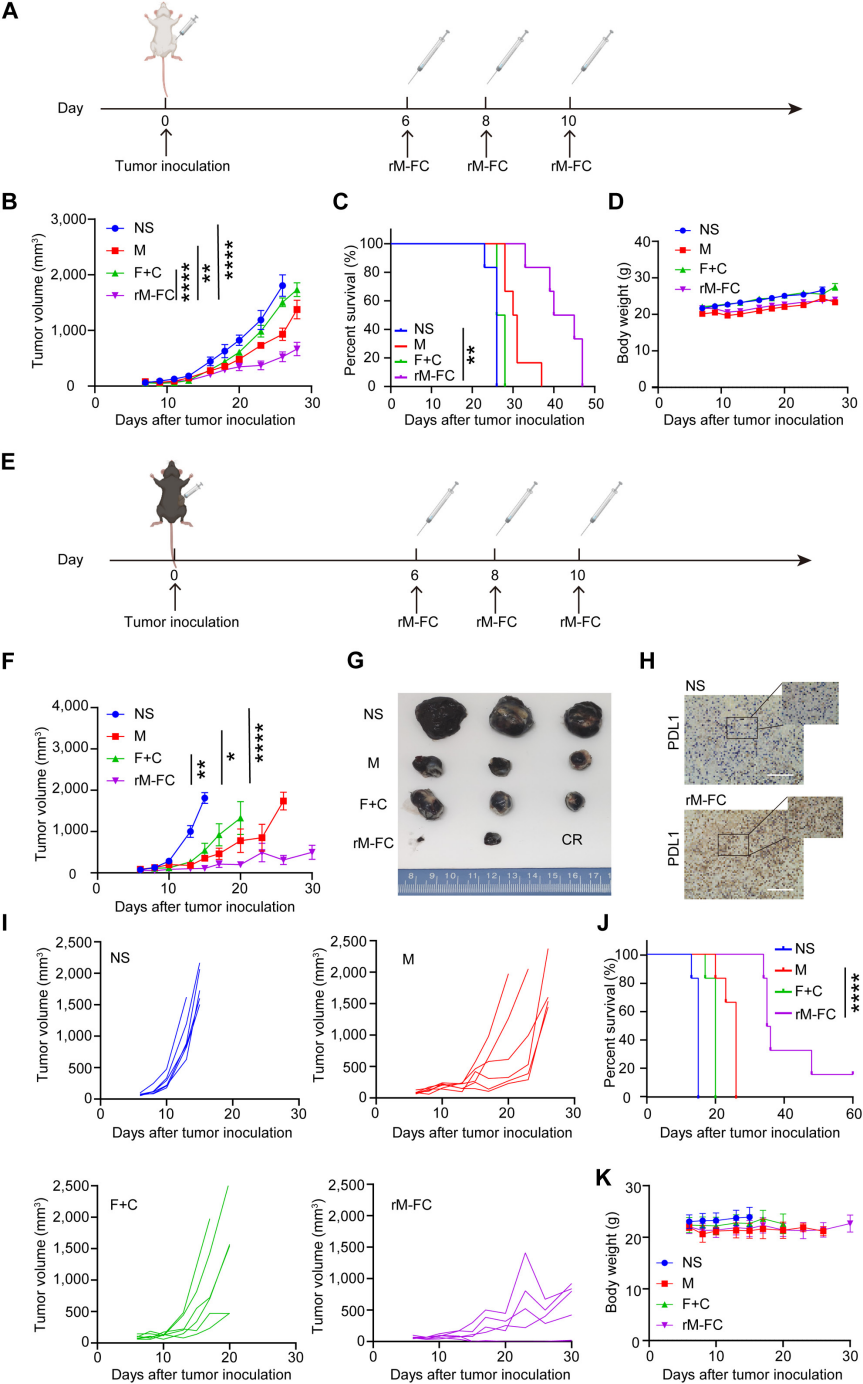
In situ vaccination inhibits established tumors. (A) Schematic diagram of intratumoral injection route of rM-FC in CT26 tumor-bearing mice. BALB/c mice were implanted with CT26 cells (1 × 10^6^) subcutaneously on the left lower sides of the abdomen on day 0 and received treatments on days 6, 8, and 10. When the tumor volume reached about 75 mm^3^, mice were randomly divided into 4 groups as follows: NS (normal saline), M (1 × 10^8^ CFU wild-type *M. smegmatis*), F+C (30 μg of Flt3L + 50 μg of CD40darpin), and rM-FC (1 × 10^8^ CFU). All drugs were dissolved in 100 μl of NS for intratumoral injection. (B) Average tumor-growth curves, (C) body weight, and (D) survival data of BALB/c mice bearing CT26 tumor with different treatments as indicated (*n* = 6). (E) Schematic diagram of intratumoral injection route of rM-FC in the B16F10 tumor suppression experiment. C57BL/6 mice were challenged with 1 × 10^5^ B16F10 tumor cells, and the dosing regimen was consistent with the above. (F) Average tumor-growth curves of C57BL/6 mice bearing B16F10 melanoma tumors with different treatments as indicated. (G) Representative photos of tumors harvested from C57BL/6 mice in all groups on the 10th day after treatments (*n* = 3). (H) Immunohistochemistry analysis of PDL1 expression in C57BL/6 treated tumors 10 d after treatments (*n* = 3). (I) Tumor-growth curves of each C57BL/6 mouse in different groups (*n* = 6). (J) Survival data of C57BL/6 mice in different groups for 60 d (*n* = 6). (K) Average body weight of C57BL/6 mice in different groups for 30 d (*n* = 6). The error bars represent mean ± SEM. For the experiments in (B) and (F), the error bars represent mean ± SEM. *P* values were calculated by 2-way analysis of variance (ANOVA) and Tukey posttest and correction. * represents *P* < 0.05, ** represents *P* < 0.01, and **** represents *P* < 0.0001. For the experiments in (D) and (K), the error bars represent mean ± SEM. Differences in survival were determined by using the Kaplan–Meier method, and the *P* value was calculated via the log-rank (Mantel–Cox) test. ** represents *P* < 0.01, and **** represents P < 0.0001.

Similar to the previous experiment of dose exploration, mice were challenged with 1 × 10^6^ CT26 tumor cells subcutaneously. When the tumor volume reached about 75 mm^3^, mice were randomly divided into 4 groups as follows: NS, M (1 × 10^8^ CFU), F+C (30 μg of Flt3L + 50 μg of CD40darpin), and rM-FC (1 × 10^8^ CFU). All drugs were dissolved in 100 μl of NS for intratumoral injection. Mice were treated at a 2-d interval after the first injection, and a total of 3 doses were administered. Notably, tumors grew at a rapid rate in the NS group and all of the mice died within 26 d. At the same time, mice treated with M and F+C showed a modest antitumor efficacy compared to the NS group (Fig. 3B). In sharp contrast, we found that rM-FC significantly suppressed tumor growth and extended the median survival time of tumor-bearing mice to 41 d (Fig. 3B and C). The body weight change remained roughly the same in all groups during the treatment (Fig. 3D).

### Intratumoral injection of rM-FC induced tumor regression in the murine B16F10 melanoma tumor model

To verify the therapeutic potential of rM-FC in “cold” tumors with poor immunogenicity, we used the malignant melanoma B16F10 tumor as an animal model. Mice were challenged with 1 × 10^5^ B16F10 tumor cells, and the dosing regimen was consistent with the previous experiment (Fig. 3E). The results showed that rM-FC also had a significant inhibitory effect on B16F10 tumors (Fig. 3F). We found that all mice in the NS group died within 16 d. In contrast, mice treated with rM-FC had slower tumor growth, and one mouse achieved complete regression. The significant antitumor effect elicited a 17% survival rate of tumor-bearing mice over 60 d (*n* = 6, Fig. 3J). Mice in different groups did not experience abnormal weight fluctuations (Fig. 3K). We further collected the treated B16F10 tumors and evaluated the change in PDL1 expression by immunohistochemical analysis. As shown in Fig. 3H, the number of PDL1 significantly increased in the TME 10 d after the injection (intratumoral) of rM-FC, which provided the rationality of the synergistic effect of rM-FC and PDL1.

### Intratumoral injection of rM-FC reprogrammed the immune microenvironment in the treated tumors and TDLNs

**Fig. 4.**
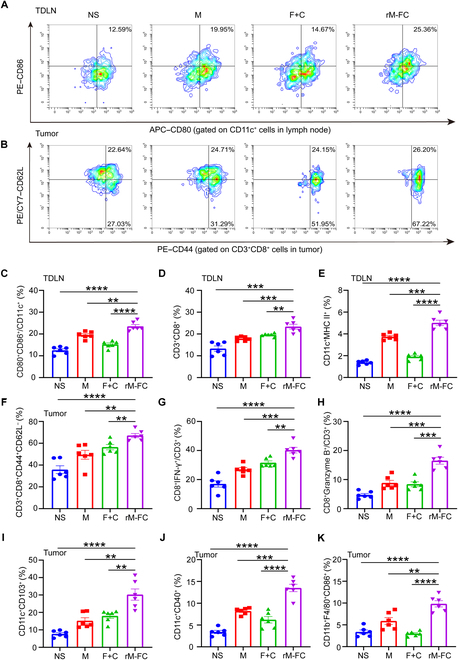
Immune response induced by the rM-FC. B16F10 tumor-bearing mice were randomly divided into 4 groups and received the same treatment regimen as described above. Ten days after the last treatment, these mice were sacrificed with their tumors and TDLNs were collected to analyze the changes in immune cells by flow cytometry. Representative flow cytometry images of (A) mDCs (CD11c^+^CD80^+^CD86^+^, gated on CD11c^+^ cells) in TDLNs and (B) central memory T cell (T_CM_; CD3^+^CD8^+^CD44^+^CD62L^+^, gated on CD3^+^CD8^+^ cells) and effector memory T cells (T_EM_; CD3^+^CD8^+^CD44^+^CD62L^−^, gated on CD3^+^CD8^+^ cells) in treated tumors. Percentage of (C) mDCs (gated on CD11c^+^ cells), (D) CD3^+^CD8^+^ cells (gated on CD3^+^ cells), and (E) CD11c^+^MHC II^+^ cells (gated on CD11c^+^ cells) in lymph nodes. Percentage of (F) T_EM_, (G) CD8^+^IFN-γ^+^ cells (gated on CD3^+^ cells), and (H) CD8^+^Granzyme B^+^ cells (gated on CD3^+^ cells) in tumors. Percentage of (I) CD11c^+^CD103^+^ cells (gated on CD11c^+^ cells) and (J) CD11c^+^CD40^+^ cells (gated on CD11c^+^ cells) in tumors. Percentage of (K) M1-like macrophages (gated on CD11b^+^F4/80^+^ macrophages in the tumor analyzed by flow cytometry after the last administration for 10 d (*n* = 6). For the experiments in (C) to (K), the error bars represent mean ± SEM. *P* values were calculated by 2-tailed unpaired Student *t* tests. ** represents *P* < 0.01, *** represents *P* < 0.001, and **** represents *P* < 0.0001.

In the previous biodistribution experiments, we found that rM-FC mainly colonized in the treated tumors and TDLNs and could be used as an effective antitumor strategy. Thus, we assumed that rM-FC might reprogram the immune microenvironment in the treated tumors and TDLNs. To assess the detailed immune response underlying the effectiveness of rM-FC treatment, B16F10 tumor-bearing mice were randomly divided into 4 groups and received the same treatment regimen as described above. Ten days after the last treatment, these mice were sacrificed with their tumors and TDLNs were collected to analyze the changes in immune cells by flow cytometry. DCs could capture and process antigens to T cells, promoting the proliferation and activation of T cells for eliciting subsequent immune responses in the TDLNs [34]. We observed a marked enhancement in the maturation of DCs after coincubating rM-FC with DCs in vitro, indicating that rM-FC had an immunostimulatory function. By analyzing the maturation status of DCs within the TDLNs in vivo, we found that effective DC maturation could be achieved after intratumoral injection of rM-FC. Compared to M, rM-FC could stimulate DC maturation more effectively. The proportion of mature dendritic cells (CD11c^+^CD80^+^CD86^+^) in the rM-FC group had the highest level, showing a 3-fold increase compared to the NS group (Fig. 4A and C). In addition, we observed that intratumoral injection of rM-FC could significantly promote the activation of both CD8^+^ T cells and MHC II^+^ DCs in TDLNs (Fig. 4D and E).

Next, changes in the TME caused by rM-FC were also evaluated. It has been reported that the effector memory T cells (T_EM_, CD3^+^CD8^+^CD44^+^CD62L^−^) play an important role in killing tumor cells [35]. Consistently, the results showed that the proportion of T_EM_ in the rM-FC-treated tumors was higher than that in the M-treated ones (Fig. 4B and F). The average proportion of T_EM_ in the M group and the rM-FC group increased from 48.28% to 65.71% (Fig. 4F), while there was no significant difference in central memory T cells (T_CM_, CD3^+^CD8^+^CD44^+^CD62L^+^) between the 2 groups (Fig. S5A). Since the functional activity of CD8^+^ T cells in the TME is of great significance, we also analyzed intracellular cytokine IFN-γ and Granzyme B in T cells. It was shown that the proportions of CD8^+^IFN-γ^+^ cells and CD8^+^Granzyme B^+^ cells were the highest in the rM-FC group (Fig. 4G and H). cDC1s were essential for the initiation and expansion of tumor-specific T cells. However, they are seriously lacking in the TME. We found that intratumoral injection of rM-FC markedly increased cDC1s such as CD103^+^ DCs in the TME. Notably, the highest proportion of CD103^+^ DCs was observed in the rM-FC group, which was 10 times as much as that in the NS group (Fig. 4I). Similarly, we found that rM-FC could upregulate the expression of the costimulatory molecule CD40 on DCs (Fig. 4J).

Furthermore, it was found that intratumoral injection of rM-FC could also trigger effective antitumor immunity by promoting frequencies of M1-like macrophages in the TME, while inhibiting frequencies of M2-like macrophages. As shown in Fig. 4K, treatment with rM-FC induced greater expression of M1-like macrophages (CD11b^+^F4/80^+^CD86^+^) and had a nearly 3-fold increase compared to that in the NS group. The proportion of M2-like macrophage (CD11b^+^F4/80^+^CD206^+^) showed no significant difference among all groups (Fig. S5B). This indicated that such an in situ vaccination could promote the transformation of M2-like macrophages to M1-like macrophages, which in turn played a role in reprogramming the TME and preventing tumor immune evasion.

Furthermore, we also detected the secretion levels of diverse cytokines in the TME. The results showed that while IL-2, IL-4, IL-5, and tumor necrosis factor-α (TNF-α) secretion remained unchanged, the secretion level of IFN-γ increased more than 4-fold compared to that in the NS group (Fig. S6).

Similar to the construction of rM-FC, we designed the engineered *M. smegmatis* expressing Flt3L (rM-F) or CD40darpin (rM-C). The successful expression of the protein was confirmed by WB (Fig. S7A and B). OVA-expressing B16F10 melanoma mouse models were used to explore the initiation of tumor-specific immune responses. Lymphocytes were isolated from TDLNs 5 d after the last treatment to examine the generation of model tumor antigen (OVA)-specific cytotoxic T lymphocytes by staining with the OVA/H-2Kb tetramer. Importantly, rM-FC generated higher antigen-specific CD8^+^ T cell responses than rM-F alone or rM-C alone, which demonstrated the necessity of the combination of Flt3L and CD40darpin to induce antigen-specific CD8^+^ T cell activation (Fig. S7C and D).

### Intratumoral injection of rM-FC induced regression of untreated distant tumors

**Fig. 5.**
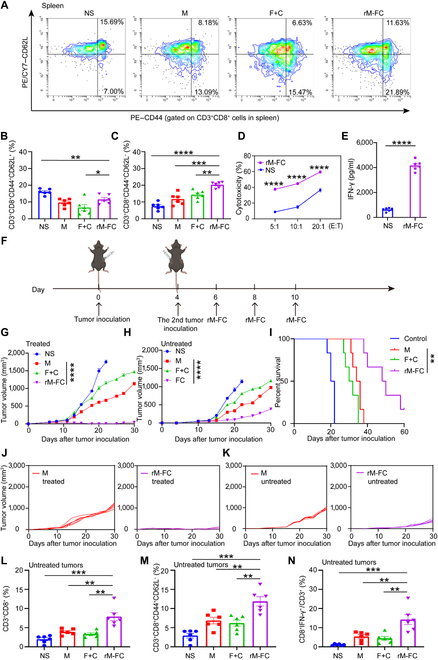
Immune memory induced by intratumoral injection of rM-FC. (A) Representative flow cytometry images of T_CM_ and T_EM_ in spleens. Percentages of (B) T_CM_ and (C) T_EM_ in spleens (*n* = 6). (D) The cytotoxic effects of splenocytes on B16F10-luc cells (*n* = 6). Splenocytes of mice in the NS or rM-FC group were incubated with B16F10-luc tumor cells at effector-to-target ratios (E:T) of 5:1, 10:1, and 20:1. Luminescence was detected by the addition of d-luciferin to B16F10-luc after incubated with splenocytes for 24 h. Cytotoxicity % = luminescence of B16F10-luc tumor cells after incubation with splenocytes of mice for 24 h/initial luminescence of B16F10-luc tumor cells. (E) The concentration of IFN-γ secreted in the supernatant of splenocytes of mice at an E:T of 20:1 (*n* = 6). (F) Schematic illustration of the experimental schedule for evaluating the abscopal effect of rM-FC on distant tumors. Average tumor-growth curves of (G) the treated primary and (H) untreated distant tumors of B16F10 melanoma tumor-bearing mice post different treatments as indicated (*n* = 6). (I) Survival data of C57BL/6 mice in different groups for 60 d (*n* = 6). (J and K) Tumor-growth curves of each mouse following the treatment (*n* = 6). Percentages of (L) CD3^+^CD8^+^ cells, (M) T_EM_, and (N) CD8^+^IFN-γ^+^ cells (gated on CD3^+^ cells) in the untreated tumors (*n* = 6). For the experiments in (B) to (E) and (L) to (N), the error bars represent mean ± SEM. *P* values were calculated by 2-tailed unpaired Student *t* tests. * represents *P* < 0.05, ** represents *P* < 0.01, *** represents *P* < 0.001, and **** represents *P* < 0.0001. For the experiments in (G) and (H), the error bars represent mean ± SEM. *P* values were calculated by 2-way ANOVA and Tukey posttest and correction. **** represents *P* < 0.0001. Differences in survival were determined by using the Kaplan–Meier method, and the *P* value was calculated via the log-rank (Mantel–Cox) test. ** represents *P* < 0.01.

The spleen contains a large number of lymphocytes that play an important role in cellular and humoral immunity. Therefore, we investigated the changes in T_CM_ and T_EM_ in the spleen. It was found that the ratio of T_CM_ and T_EM_ was elevated in the rM-FC group compared to that in the NS group (Fig. 5A to C). In order to study the tumor-killing ability produced by rM-FC under this program, we incubated splenocytes of the rM-FC-treated mice with B16F10 melanoma cells ex vivo at different ratios. Compared to the those of the NS group, the splenocytes of the rM-FC-treated mice exhibited a stronger cytotoxic activity (Fig. 5D). Consistently, the level of IFN-γ, which is secreted by splenocytes, also increased in the rM-FC group (Fig. 5E). Notably, these results demonstrated that intratumoral injection of rM-FC induced a potent tumor killing, which was essential for the long-term prevention of tumor recurrence. We further determined whether intratumoral injection of rM-FC could generate immune memory to produce systemic antitumor immunity in vivo. The abscopal effect of rM-FC was examined in male mice bearing 2 B16F10 tumors (Fig. 5F). In contrast to the other groups, intratumoral injection of rM-FC inhibited the growth of not only the treated tumors but also the untreated distant ones (Fig. 5G, H, J, and K). Furthermore, the percent survival in the rM-FC group was significantly longer than that in the M group (Fig. 5I). It was worth noting that we did not find the bacteria in distant untreated tumors in the previous experiment (Fig. S2A to C), suggesting that the regression of distant untreated tumors was due to systemic immunity induced by the engineered bacteria, rather than the migration of rM-FC. It is significant to evaluate the immunological phenotypes in the untreated site. Compared to the NS group, the proportion of CD3^+^CD8^+^ T cells, T_EM_ (CD3^+^CD8^+^CD44^+^CD62L^−^) subset, and IFN-γ^+^ CD8^+^ T cells increased at the untreated site 10 d later in the rM-FC group (Fig. 5L to N).

### Combination therapy of rM-FC with anti-PDL1 had a synergistic antitumor effect and generated a long-term immune memory effect

**Fig. 6.**
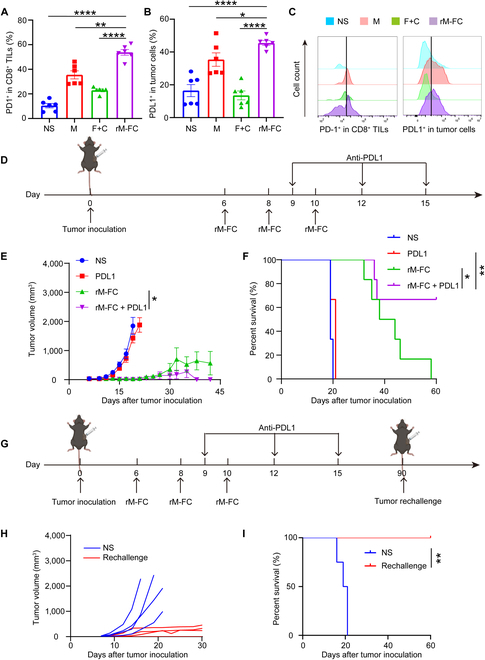
Combination therapy of rM-FC with anti-PDL1. The expression of (A) PD-1 on CD8^+^ tumor-infiltrating T lymphocytes (TILs) and (B) PDL1 on tumor cells of C57BL/6 mice in different groups after the last administration for 10 d (*n* = 6). (C) Representative flow cytometry images of PD-1 on CD8^+^ TILs and PDL1 on tumor cells of C57BL/6 mice in different groups after the last administration for 10 d. (D) Schematic diagram of the administration route for rM-FC combined with anti-PDL1 therapy. (E) Average tumor-growth curves of C57BL/6 mice bearing B16F10 melanoma tumor with different treatments as indicated (*n* = 6). (F) Survival data of C57BL/6 mice in different groups for 60 d (*n* = 6). (G) Schematic illustration of evaluating the immune memory effect of rM-FC on the rechallenged tumors. Mice cured of B16F10 tumors were rechallenged subcutaneously 90 d later with the same number of tumor cells (1 × 10^5^) at the side of the abdomen. (H) Tumor growth curves of each mouse following the rechallenge (*n* = 4). (I) Survival data of 2 groups of mice (*n* = 4). For the experiments in (A) and (B), the error bars represent mean ± SEM. *P* values were calculated by 2-tailed unpaired Student *t* tests. * represents *P* < 0.05, ** represents *P* < 0.01, and **** represents *P* < 0.0001. For the experiments in (E), the error bars represent mean ± SEM. *P* values were calculated by 2-way ANOVA and Tukey posttest and correction. * represents *P* < 0.05. Differences in survival were determined by using the Kaplan–Meier method, and the *P* value was calculated via the log-rank (Mantel–Cox) test. * represents *P* < 0.05 and ** represents *P* < 0.01.

Although rM-FC could effectively inhibit tumor growth in CT26-bearing and B16F10-bearing mice, the tumor was still growing at a slow rate within 30 d after the treatment with rM-FC. Only a few tumor-bearing mice could achieve complete regression eventually. In the previous experiments, we observed a remarkable increase in the expression of PD1 on CD8^+^ tumor infiltration T lymphocytes and PDL1 on the tumor cells in the rM-FC group compared to that in the NS group (Figs. 3H and 6A to C). While activating T cells and effectively initiating the immune response, rM-FC treatment increases the expression of PDL1^+^ tumor cells, which transmit inhibitory signals. PD-L1 inhibitors block PD-L1 signaling, having the potential to reactivate T cells to recognize and attack tumor cells, and generate an immune memory effect to prevent tumor recurrence.

To investigate whether PD-L1 blockade could improve antitumor immune responses in combination with rM-FC, rM-FC-treated mice received anti-PDL1 intraperitoneally every third day at a dose of 200 μg/mouse (Fig. 6D). We observed that combination therapy of rM-FC with anti-PDL1 could trigger a significant regression of tumor growth in B16F10 tumor-bearing mice compared to the mice treated only with rM-FC (Fig. 6E). Meanwhile, NS-treated and PDL1-treated mice all died within 21 d after tumor inoculation, while only 2 mice in the rM-FC and anti-PDL1 combination treatment group died within 40 d (*n* = 6), and the mouse survival rate increased to 67% (Fig. 6F).

To further investigate whether the combination therapy could induce an immunological memory effect to prevent tumor recurrence, we rechallenged the cured mice with 1 × 10^5^ B16F10 tumor cells 90 d after the last treatment (Fig. 6G). It was surprising to find that the tumor-growth rate of these cured mice was significantly slower than that in the untreated group, and one mouse completely rejected the recurrence of the tumor (Fig. 6H). All cured mice survived for more than 30 d (Fig. 6I). These results provided the potential of combining rM-FC with anti-PDL1 in future clinical translation.

### Biosafety assessment of rM-FC

**Fig. 7.**
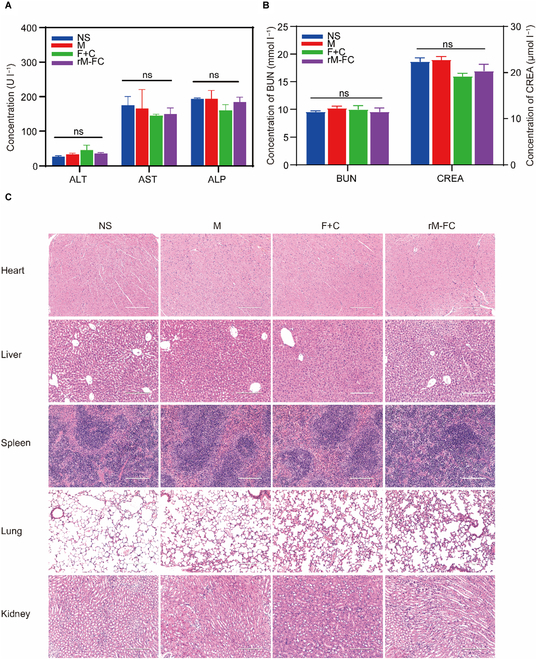
Biosafety assessment of rM-FC. Blood biochemistry of the (A) liver function (alanine aminotransferase [ALT], aspartate aminotransferase [AST], and alkaline phosphatase [ALP]) and (B) kidney function (blood urea nitrogen [BUN] and creatinine [CREA]) analysis of B16F10 tumor-bearing mice intratumoral injection with 100 μl of NS, M (1 × 10^8^ CFU), F+C (30 μg of Flt3L + 50 μg of CD40darpin), and rM-FC (1 × 10^8^ CFU) (*n* = 3). The error bars represent mean ± SEM. *P* values were calculated by 2-tailed unpaired Student *t* tests. ns represents *P* > 0.05. (C) Hematoxylin–eosin staining of main organs, namely, the heart, liver, spleen, lung, and kidney, in B16F10 tumor-bearing mice. The scale bar is 200 μm.

Intratumoral injection of live bacteria, even these nonpathogenic bacteria, is prone to causing some side effects, such as cytokine storms, body weight dropping, and even death. When rM-FC was intratumorally injected, the body weight of CT26 tumor-bearing mice and B16F10 tumor-bearing mice did not fluctuate significantly compared to the mice treated with the NS group (Fig. 3D and K). By examining the liver and kidney function serum biochemistry indicators (alanine aminotransferase, aspartate aminotransferase, alkaline phosphatase, blood urea nitrogen, and creatinine) of C57BL/6 mice in different treatment groups to assess the systemic inflammatory response, we found that there was no significant difference in the serum biochemistry indexes of the mice in 4 groups and all these indicators were within the normal range (Fig. 7A and B). Furthermore, hematoxylin and eosin staining of the main organs was performed on the 10th day after the last treatment, which did not show any difference compared with the healthy ones (Fig. 7C). Inflammatory cytokines are cytokines that regulate a variety of inflammatory responses, mainly including 2 types of pro-inflammatory cytokines (IFN-γ, TNF-α, IL-2, IL-6, etc.) and anti-inflammatory cytokines (IL-4, IL-5, IL-10, IL-13, etc.). The balance between pro-inflammatory cytokines and anti-inflammatory cytokines is essential for maintaining normal immune system function and tissue repair. When this balance is disrupted, it will lead to the development of a chronic inflammatory state. This persistent inflammatory environment not only causes localized tissue damage but can also lead to a range of systemic health problems such as cardiovascular disease, diabetes, and autoimmune diseases. Therefore, we evaluated the levels of relevant cytokines in the serum of treated mice. Consistently, the serum concentrations of IFN-γ, TNF-α, IL-2, IL-6, IL-4, IL-10, IL-5, and IL-13 were generally similar between the NS group and the rM-FC group without a statistical difference (Fig. S8). These results all showed that intratumoral injection of rM-FC had a good biological safety.

## Discussion

In this work, we have demonstrated that the nonpathogenic bacterium *M. smegmatis* has marked antitumor properties in local tumor immunotherapy models. BCG has a long history as an immunoadjuvant therapy for superficial bladder cancer and is still the only drug approved by the US Food and Drug Administration for the treatment of high-risk non-muscle-invasive bladder cancer. Although BCG is an attenuated strain, the administration of live BCG still has obvious toxicity, especially for immunodeficiency patients. *M. smegmatis*, an atypical mycobacterium, compared with the typical mycobacterium BCG, has a faster growth rate and is commensal to humans and mice. The safety and efficacy of *M. smegmatis*-based vaccines have been demonstrated in previous studies [36]. In addition, *M. smegmatis* is an excellent choice for protein production compared to other mycobacteria [27,37]. Based on the above considerations, we selected *M. smegmatis* to deliver the Flt3L and CD40darpin fusion protein as an antitumor biotherapeutic agent.

The DC-based autologous prostate cancer vaccine sipuleucel-T (Provenge) has been the first and the only DC therapeutic vaccine approved by the Food and Drug Administration so far [38]. DC vaccines have been reported to be safe and immunogenic, protecting against various cancers. However, the clinical response of DC vaccine-based immunotherapies has been disappointing. This is largely attributed to the tumor immunosuppressive microenvironment that limits the function of DCs [39]. In our study, an engineered *M. smegmatis*-based in situ vaccine has been designed to convert “cold” tumors into “hot” tumors and remodel tumor-associated macrophages to reverse the tumor immunosuppressive microenvironment. Flt3L can recruit cDC1s, especially CD103^+^ DCs, into the TME; *M. smegmatis* promotes the maturation of cDC1s; and CD40darpin can target CD40 expressed on antigen-presenting cells, thereby activating antigen-loaded cDC1s for CD8^+^ T cell initiation and expansion. It can be seen that intratumoral injection of *M. smegmatis* expressing Flt3L and CD40darpin fusion proteins can achieve a triple-positive effect on DCs while also reducing the inconvenience of multiple administrations of different DC-activating agents [19].

In clinically relevant immunotherapy, the immunosuppressive TME is the main factor in inhibiting the efficacy of related drugs, which can lead to the reduced ability of DCs to recognize antigens and their insensitivity to immune checkpoints, and thus lowers the killing efficiency of T cells [40,41]. Our data indicated that this engineered *M. smegmatis* could effectively reverse the immunosuppressed TME by inducing strong antitumor immunity, producing an abscopal effect to suppress the growth of distant untreated tumors. Two different animal models showed that the tumor inhibition effect of recombinant bacteria was far superior to those of the individual proteins. In particular, mice treated with rM-FC had significantly longer survival than those in the other group. With the help of an anti-PDL1 antibody, this engineered *M. smegmatis* could produce a powerful immune memory effect to prevent the same rechallenged tumor from growing over a long period. Furthermore, we have proved that intratumoral injection of this engineered *M. smegmatis* could effectively accumulate in treated tumors and TDLNs and was almost not found in normal tissues, showing excellent biosafety while remodeling the immune microenvironment of the treated tumors and TDLNs.

Some limitations of our study are that not enough data provide insight into specific changes in immune cells in distant untreated tumors and some tumors will relapse later in treatment, especially in tumor models with poor immunogenicity. Based on the above points, we can detect the changes in the immune microenvironment of distant tumors by flow cytometry, increasing the dose, or adjusting the frequency of administration to inhibit tumor relapse. Moreover, single-cell transcriptome analysis can be performed if necessary, which is important for understanding the mechanism of tumorigenesis.

In summary, this study highlights a potential strategy for promoting immunotherapy based on rM-FC to reshape the tumor immune microenvironment. We envisage that this engineered *M. smegmatis* has great promise for future clinical translation due to its excellent antitumor effects and acceptable safety profile.

## Ethical Approval

All animal experiments in this study were approved by the Ethics Committee of Drum Tower Hospital (Nanjing, China).

## Data Availability

The data that support the findings of this study are available from the corresponding author upon reasonable request.
