# Supplementary material for: Intratumoral Injection of Engineered Mycobacterium smegmatis Induces Antitumor Immunity and Inhibits Tumor Growth
Source: Biomater Res. 2024 Jan 7;29:0130. doi: 10.34133/bmr.0130 (PMC11704092; doi:10.34133/bmr.0130)
Supplement: Supplementary 1 — Figs. S1 to S8 [file bmr.0130.f1.docx]

Supporting Information


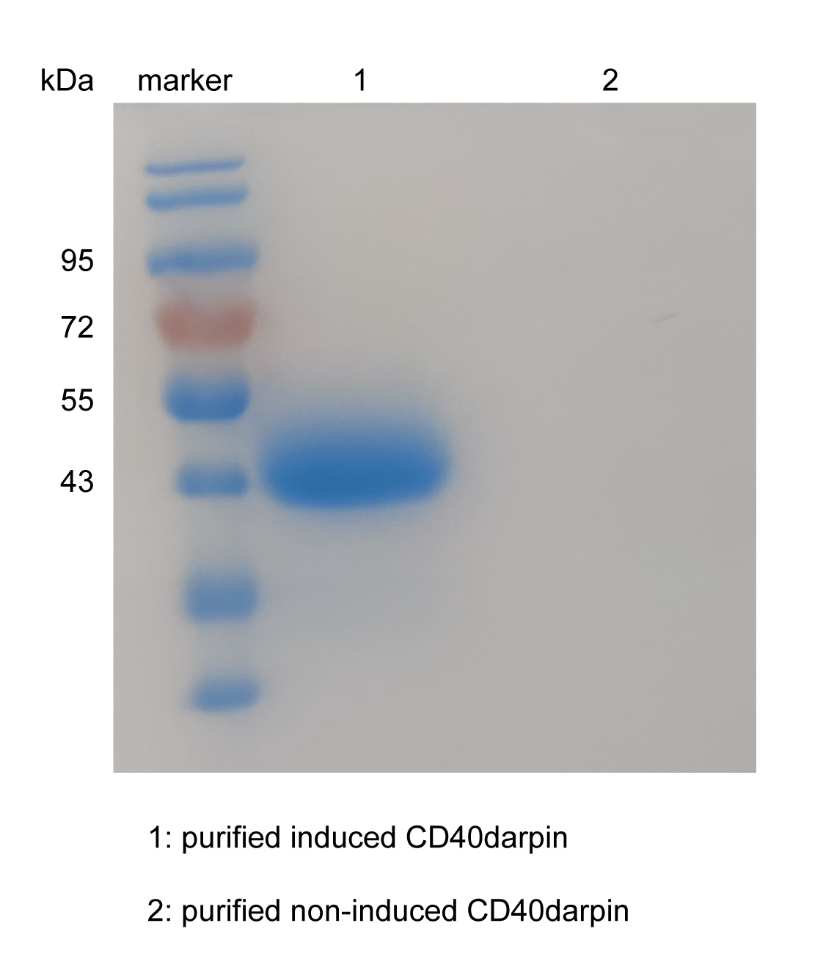


Figure S1. SDS-PAGE analysis of the induced engineered Escherichia coli. Lane marker: molecular mass marker; Lane 1: purified induced CD40darpin; Lane 2: purified non-induced CD40darpin.


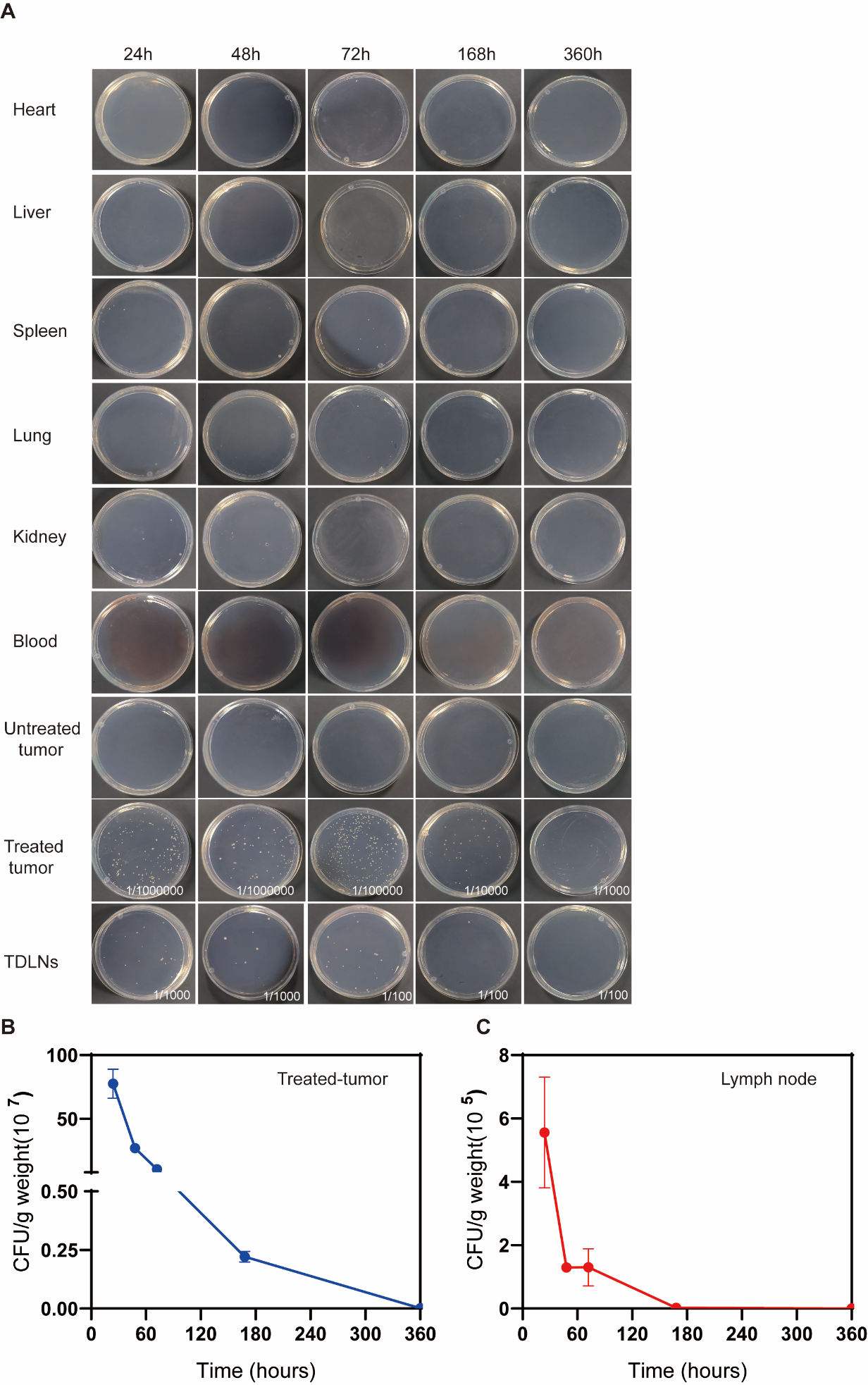


Figure S2. In vivo biodistribution of rM-FC. A) Representative photographs of rM-FC colonization in blood, hearts, livers, spleens, lungs, kidneys, treated-primary tumors, untreated-distant tumors, and TDLNs harvested from CT26-bearing mice at different time points after intratumorally injection rM-FC. Each mouse received 1×10^8^ CFU rM-FC. At 24h, 48h, 72h, 168h, and 360h post-injection, the organizations described above were collected, wet-weighted, and homogenized in 1ml sterilized PBS. Then these samples were diluted and plated on Middlebrook 7H10 agar plates and incubated at 37°C. Quantification of rM-FC colonization in B) treated-primary tumor and c) TDLNs (n=3). The error bar represented mean ± SEM.


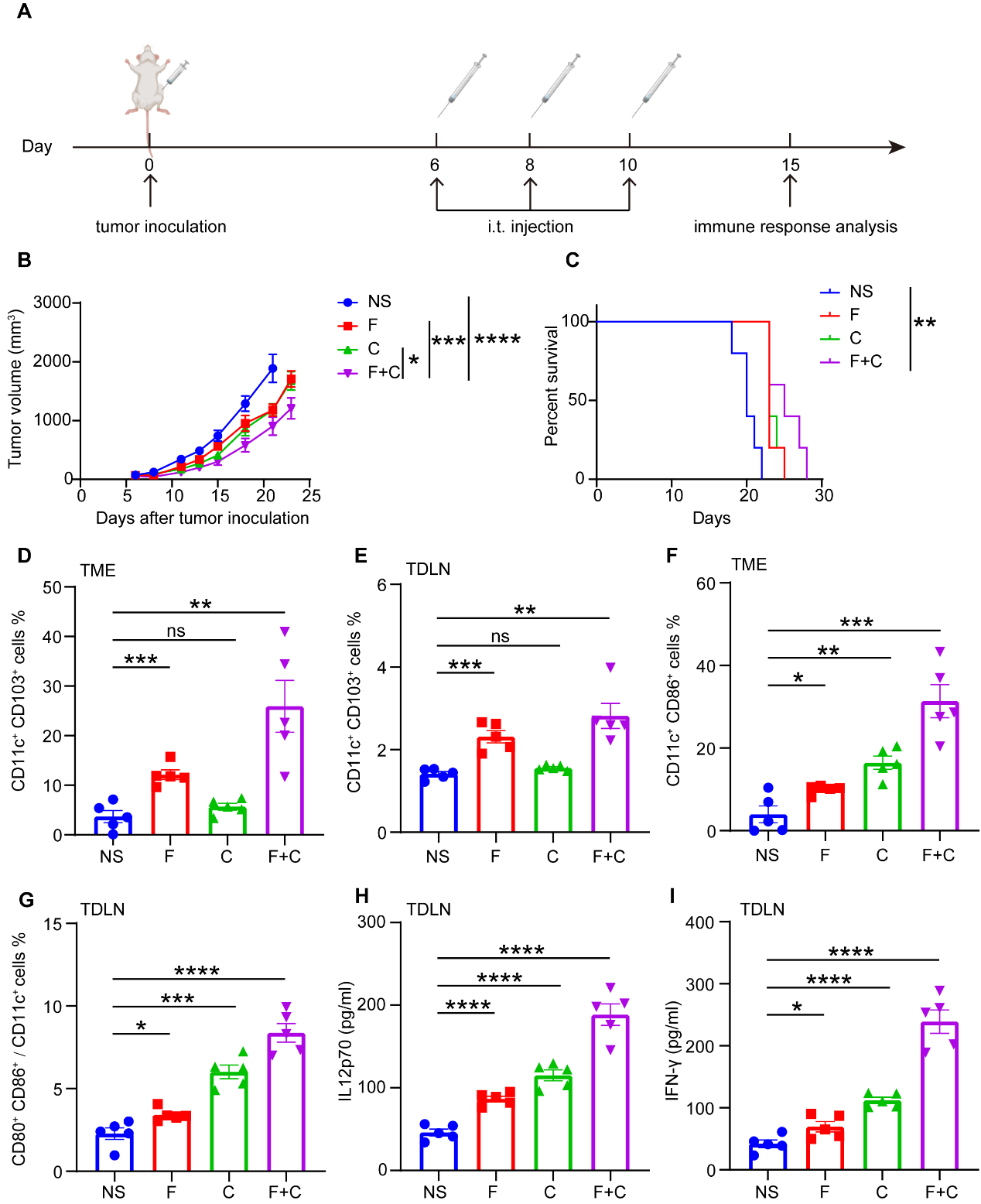


**Figure S3. Synergistic antitumor effect of Flt3L and CD40darpin. A)** Schematic diagram of intratumoral injection route of Flt3L and CD40darpin in tumor suppression experiment. **B)** Average tumor-growth curves of BALB/c mice bearing CT26-tumor with different treatments as indicated (n=5). The mice were implanted with CT26 cells (1×10^6^) on the left lower sides of the abdomen on day 0 and received treatment on days 6, 8, and 10. The immune response was analyzed on day 15. For each mouse, 30μg Flt3L and 50μg CD40darpin were dissolved in saline. The error bars represented mean ± SEM. P-values were calculated by two-way ANOVA and Tukey post-test and correction. * represented p < 0.05, and *** represented p < 0.001. **C)** Survival data of BALB/c mice in different groups for 30 days (n=5). The error bars represented mean ± SEM. P-values were calculated via the log-rank (Mantel-Cox) test. ** represented p < 0.01. **D)** Percentage of CD103^+^ DCs (gated on CD11c^+^ cells) in tumors (n=5). **E)** Percentage of CD103^+^ DCs (gated on CD11c^+^ cells) in TDLNs (n=5). **F)** Percentage of CD86^+^ DCs (gated on CD11c^+^ cells) in tumors (n=5). **G)** Percentage of CD80^+^ CD86^+^ DCs (gated on CD11c^+^ cells) in TDLNs (n=5). **H-I)** The concentration of IL12p70 and IFN-γ in TDLNs (n=5). For the experiments in **D)**-**I)**, the error bars represented mean ± SEM. P-values were calculated by two-tailed unpaired Student's t-tests. ns represented *p* > 0.05, * represented *p* < 0.05. ** represented *p* < 0.01. *** represented *p* < 0.001. **** represented *p* < 0.0001.


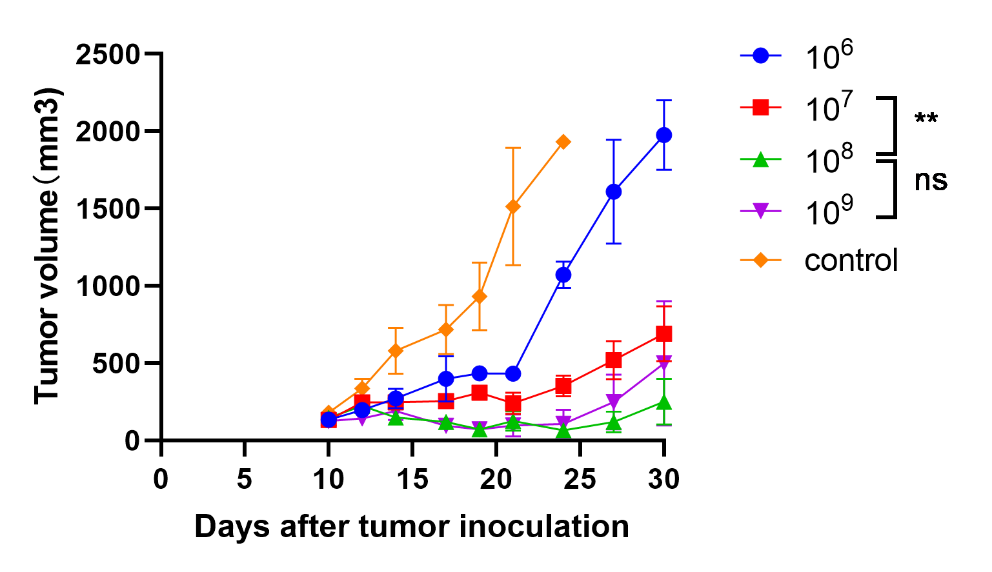


**Figure S4.** Average tumor-growth curves of BALB/c mice bearing CT26 colon tumor with different treatments as indicated (n=3). The mice were administered with NS, 10^6^CFU rM-FC, 10^7^CFU rM-FC, 10^8^CFU rM-FC, and 10^9^CFU rM-FC intratumorally when the tumor reached about 75mm^3^. The tumor size was measured every 2-3 days from the first administration day. The error bars represented mean ± SEM. P-values were calculated by two-way ANOVA and Tukey post-test and correction. ns represented p > 0.05, ** represented p < 0.01.


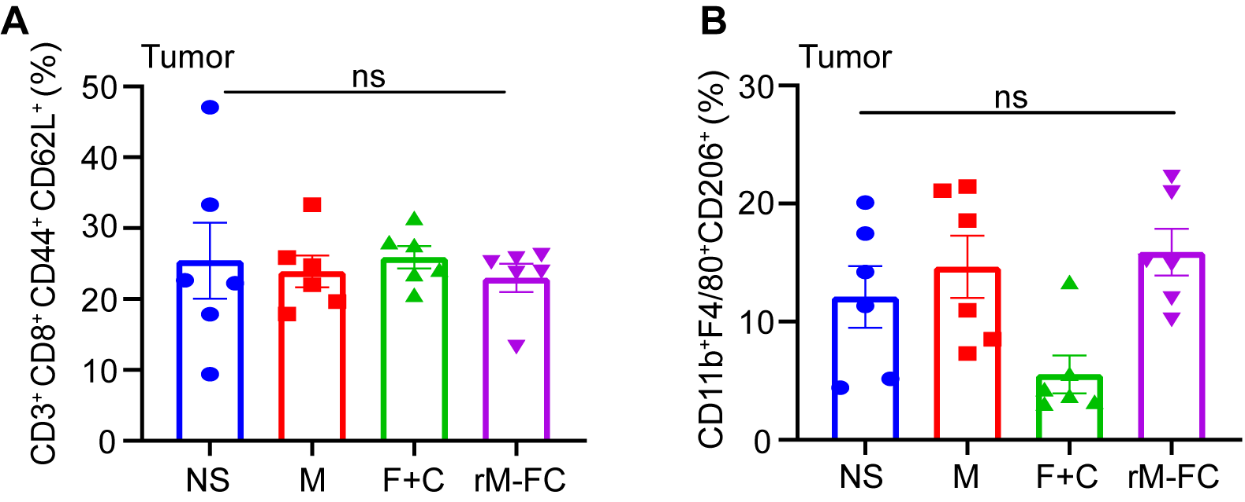


Figure S5. Immune response induced by the rM-FC. B16F10 tumor-bearing mice were randomly divided into four groups and received the same treatment regimen as described above. Ten days after the last treatment, these mice were sacrificed with their tumors, and TDLNs collected to analyze the changes in immune cells by flow cytometry. Percentage of A) T_CM_ and B) M2-like macrophages (gated on CD11b^+^ F4/80^+^ macrophages) in tumors. The error bars represented mean ± SEM. P-values were calculated by two-tailed unpaired Student’s t-tests. ns represented *p* > 0.05.


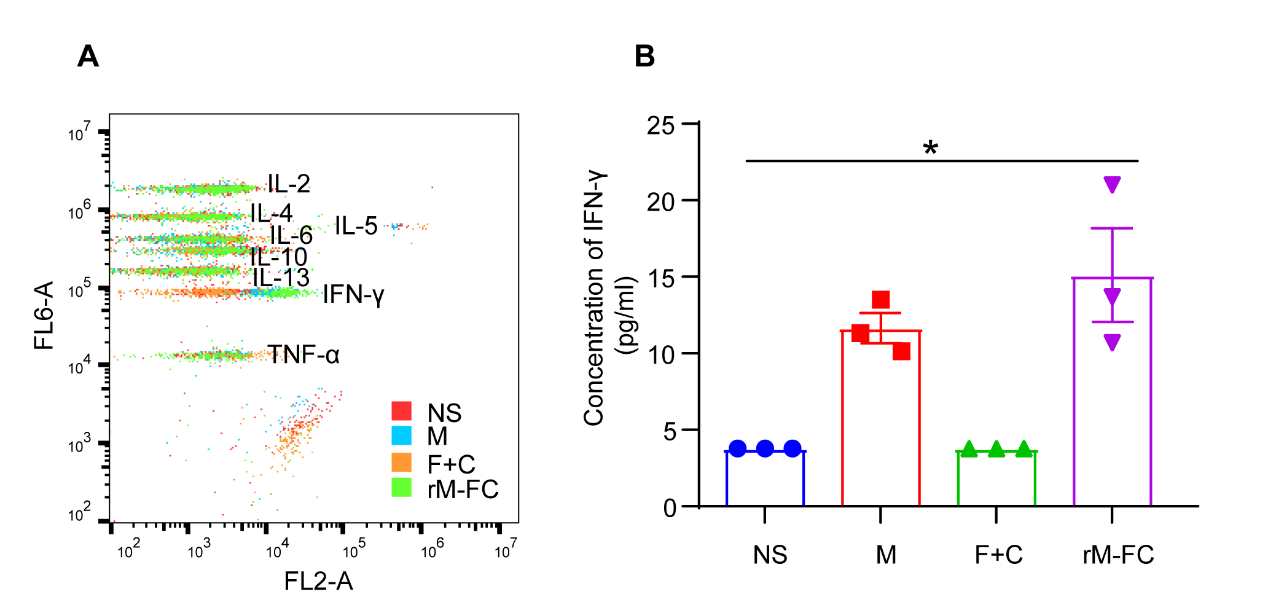


**Figure S6. A)** Representative flow cytometry images of eight cytokines (IL-2, IL-4, IL-5, IL-6, IL-10, IL-13, IFN-γ, and TNF-α) secreted in the supernatant of tumor cells of C57BL/6 mice in different group after the last administration for 10 days. **B)** The concentration of IFN-γ (n=3). The error bars represented mean ± SEM. P-values were calculated by two-tailed unpaired Student's t-tests. * represented p < 0.05.


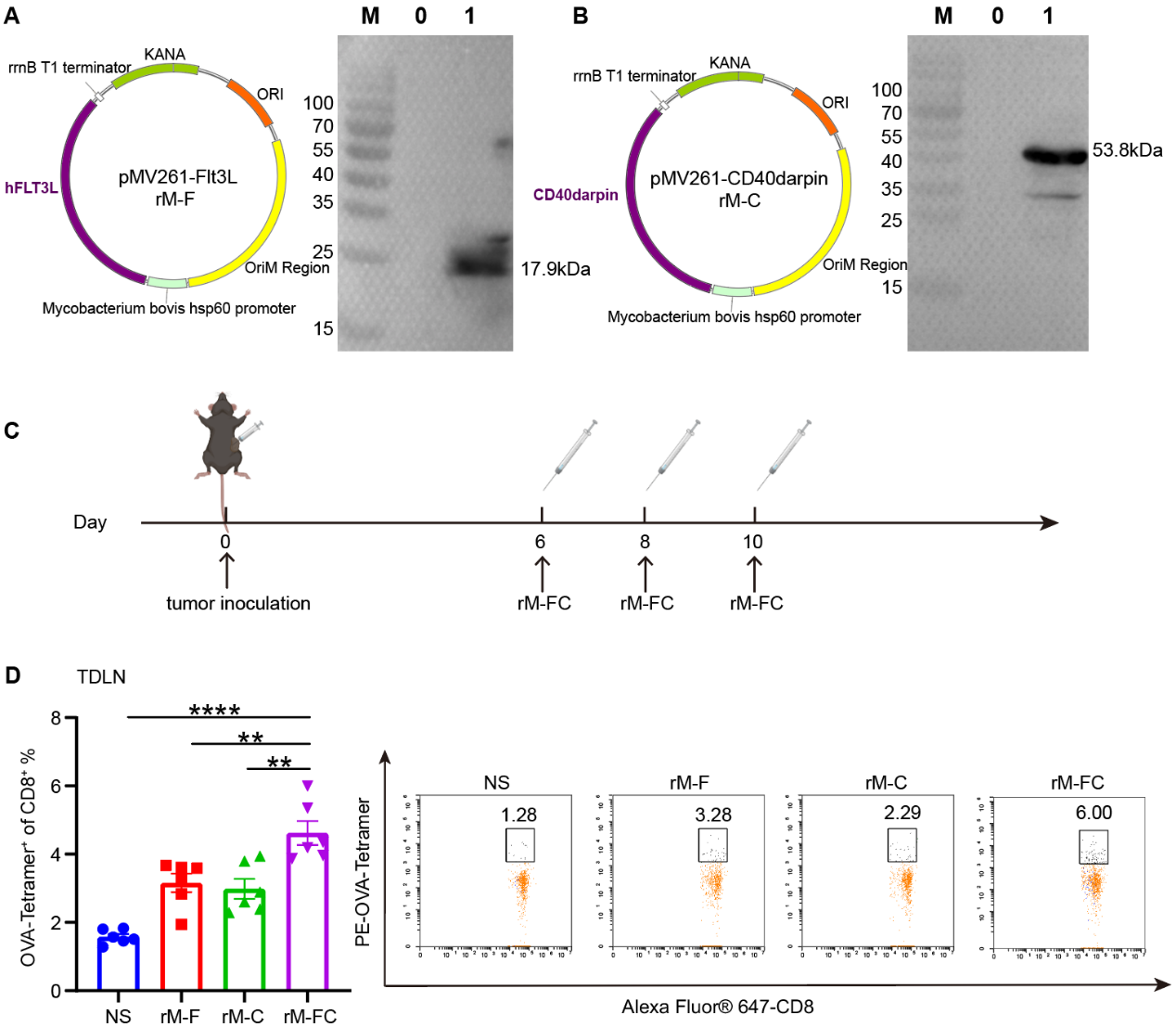


**Figure S7. The antigen-specific immune response induced by the rM-FC. A)** Left: Plasmid map of a Mycobacterium-Escherichia Coli shuttle vector using pMV261 expressing the hFlt3L. Right: Western blotting analysis of the induced engineered Mycobacterium smegmatis (rM-F). Lane marker: molecular mass marker; Lane 0: Whole bacteriological lysate of wild-type Mycobacterium smegmatis after heat shock; Lane 1: rM-F induced by heat shock. **B)** Left: Plasmid map of a Mycobacterium-Escherichia Coli shuttle vector using pMV261 expressing the CD40darpin. Right: Western blotting analysis of the induced engineered Mycobacterium smegmatis (rM-C). Lane marker: molecular mass marker; Lane 0: Whole bacteriological lysate of wild-type Mycobacterium smegmatis after heat shock; Lane 1: rM-C induced by heat shock. **C)** Schematic diagram of intratumoral injection route of rM-FC in B16F10-OVA tumor suppression experiment. C57BL/6 mice were challenged with 5 × 10^5^ B16F10-OVA tumor cells and the dosing regimen was consistent with the above. **D)** B16F10-OVA tumor-bearing mice were randomly divided into four groups and received the same treatment regimen as described above. Five days after the last treatment, these mice were sacrificed with TDLNs collected to analyze the changes in immune cells by flow cytometry (n=6). Left: Percentage of OVA-tetramer^+^ CD8^+^ T cells (gated on CD3^+^ T cells). Right: Representative flow cytometry images of OVA^+^CD8^+^ T cells gated on CD3^+^ T cells. The error bars represented mean ± SEM. P-values were calculated by two-tailed unpaired Student’s t-tests. ** represented p < 0.01. *** represented p < 0.001.


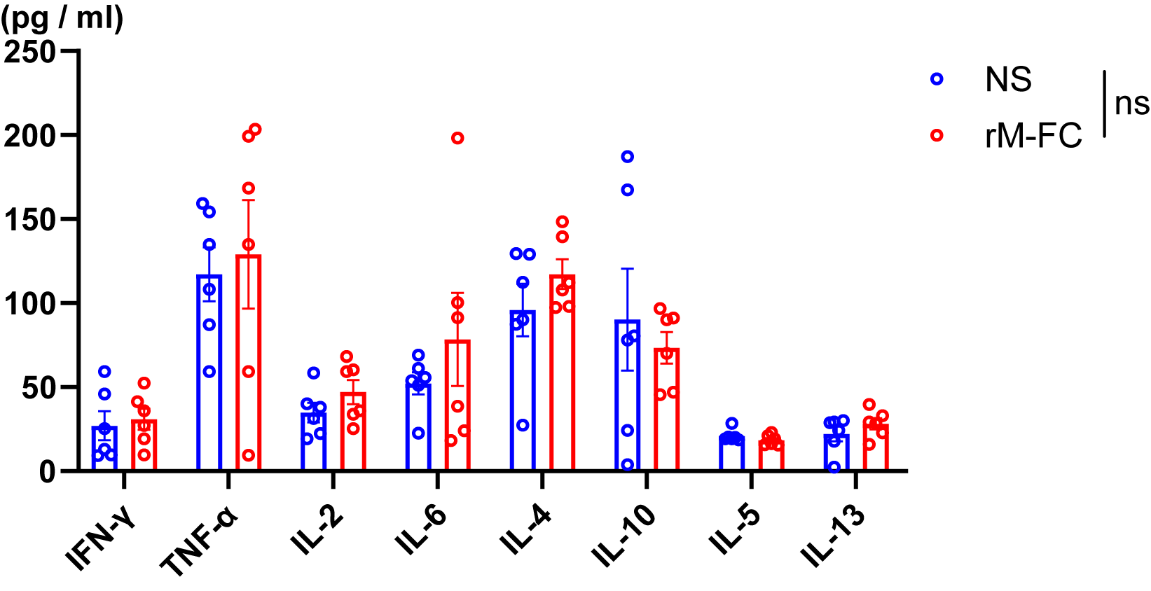


**Figure S8. rM-FC induced low systemic inflammation.** The levels of interferon-gamma (IFN-γ), TNF-α, IL-2, IL-6, IL-4, IL-10, IL-5, and IL-13 in serum from B16F10 tumor-bearing mice isolated ten days after the last treatment (n=6). The error bars represented mean ± SEM. P-values were calculated by two-tailed unpaired Student's t-tests. ns represented p > 0.05.
